# Efficacy study comparing a CBT-I developed for shift workers (CBT-I-S) to standard CBT-I (cognitive behavioural therapy for insomnia) on sleep onset latency, total sleep time, subjective sleep quality, and daytime sleepiness: study protocol for a parallel group randomised controlled trial with online therapy groups of seven sessions each

**DOI:** 10.1186/s13063-024-08403-3

**Published:** 2024-08-26

**Authors:** Tanja Grünberger, Christopher Höhn, Manuel Schabus, Anton-Rupert Laireiter

**Affiliations:** Department of Psychology, University of Salzburg, Hellbrunner Straße 34, Salzburg, 5020 Austria

**Keywords:** Insomnia, Shift workers, Efficacy study, CBT-I vs. a newly developed manual, Online group therapy

## Abstract

**Background:**

Shift workers are at an increased risk of developing sleep disorders. The standard therapy recommended for sleep disorders is cognitive behavioural therapy for insomnia (CBT-I). Many of its interventions are based on a regular sleep and wake rhythm, which is difficult to apply for shift workers. We have therefore developed a new therapy manual specifically for shift workers (CBT-I-S), which should be more applicable to their needs. In particular, all interventions that require regularity have been removed, and instead, interventions that address factors that proved to be relevant to sleep in our preliminary study have been integrated. We now want to test this manual for its effectiveness.

**Methods:**

A randomised controlled trial with *N* = 142 will be conducted to compare two conditions: the newly developed therapy manual will be carried out in the experimental group, while cognitive behavioural therapy for insomnia will be employed in the standard group. Both treatments will be conducted online via MS Teams in a group setting with seven sessions each. Data will be collected at three measurement points (pre, post, 6-month follow-up) and analysed using linear mixed models. The study will investigate whether the two treatments have led to significant improvements in total sleep time, sleep onset latency, subjective sleep quality and daytime sleepiness in shift workers. It will also examine whether the new therapy manual is superior to standard therapy in shift workers and whether these effects are stable.

**Discussion:**

We assume that interventions designed to address depressive mood, anxiety, worry, rumination, dysfunctional thought patterns and attitudes towards sleep will also improve sleep. If this is indeed the case, these interventions could replace previous ones that require regularity. This could significantly improve the treatment of insomnia in shift workers.

**Trial registration:**

German Clinical Trials Registry DRKS DRKS00032086. Registered on August 16, 2023.

## Introduction

### Background and rationale {6}

The current state of research on the subject of sleep or sleep disorders and their therapy is already well advanced, see various textbooks and standard works (e.g., [1–6]). In most of these studies, however, shift workers are explicitly excluded, even though this group in particular has an increased risk of sleep disorders. Reynolds et al. [7] estimate the proportion of shift workers at 20% of all employees, of whom 20–40% could suffer from clinically relevant sleep disorders.

According to Nachreiner et al. [8], shift work leads to a desynchronization of working time with both endogenous circadian and exogenous social rhythms resulting into impairments in sleep, cognitive functions, performance, and health, but also social impairments for those affected and their social environment ([8], p. 204).

Costa [9] identified the effects of shift work on sleep as a reduction in total sleep time by 2 to 4 h, an increase in the proportion of REM and stage 2 sleep, which is perceived as less restful, and more frequent and/or earlier sleep interruptions, partly for social reasons, for example to eat with the family. According to Seibt et al. [10], this is not only due to chronobiology, but also to the more difficult sleeping conditions during the day, mainly due to brightness and higher noise levels.

Sleep disorders in shift workers are listed in DSM-5 [11, 12] under 307.45, circadian rhythm disorder, shift work type. According to Drake et al. [13], one of the following criteria is present: insomnia and/or excessive sleepiness associated with working hours that fall within the usual sleep times.

The gold standard of insomnia treatment is CBT-I (cognitive behavioural therapy for insomnia). It mainly consists of psychoeducation, and cognitive restructuring of dysfunctional assumptions about sleep as well as training subjects in sleep hygiene, sleep restriction, stimulus control, and relaxation, [7, 14]. However, because these interventions require regular sleep and wake times, they are not suitable for shiftworkers [7]. In a meta-analysis [7], 10 studies were examined in which the CBT-I was partly carried out in a slightly modified form with shift workers. In some cases, however, very specific target groups were used, such as firefighters [15] or media workers [16]. Some of these studies require caution in interpreting the results, either because of a very small sample size (e.g., *N* = 19, [16]) or because of small effects [17] or lacking a control group. Sometimes non-psychological methods were used, such as a sports programme [18]. Summarising the results of studies of CBT-I in shift workers, Reynolds et al. [7] conclude that although there is a reduction in symptoms as a result of these interventions, on average this reduction does not reach clinically significant levels. They see this as evidence that the use of standard CBT-I is not appropriate for this target group.

Referring to the studies by Booker et al. [17] and Lee et al. [19], Ito-Masui et al. [20] conclude that CBT-I adapted to shift workers is effective. In their own study [20], they conducted a blended online intervention consisting of an adapted, app-supported, clinician-guided therapy. Although the results showed statistically significant improvements, they were only partially clinically significant. In addition, they did not use a control group design, and the sample consisted almost entirely of female nurses, which raises issues about the generalisability of the results.

The current state of research on the effectiveness of CBT-I for shift workers is therefore limited or suggests that previous approaches are not sufficiently effective. A recent study [21] shows very encouraging results for a digital CBT-I adapted to shift workers, although it was only tested on a waiting list control group. Another recent study [22] differentiates between daytime and night-time insomnia and has developed corresponding treatment approaches that have also led to good effects. Here, too, only a passive control group is used.

We will therefore conduct a study that takes these points of criticism into account. The contents of the manual used in this study were derived from a literature analysis and an own preliminary study that has not yet been published. Interventions that require regularity have been replaced. Alternative appointments in parallel groups as well as missing appointments are possible. In the latter case, for compensation subjects can read the content themselves online or watch it as a video.

An exaggerated focus on disturbed sleep is a strong supporting factor of the sleep disorder, which can lead to an intention to force falling asleep and thus prevent falling asleep (attention-intention-effort pathway, [23]). The newly developed manual for shift workers (CBT-I-S) derives its therapeutic rationale from this model: The focus of attention should be shifted away from (the disturbed) sleep and, implicitly, sleep should be improved. To this end, interventions are applied to other factors influencing sleep that have been shown to be relevant in our own preliminary study: depressed mood, anxiety, worrying, rumination, dysfunctional thought patterns and attitudes towards sleep. The interventions used have their primary source in behavioural and positive psychology.

In our preliminary study, attitudes to shift work were another important factor affecting sleep. Cognitive restructuring was used to help participants develop a more positive attitude towards shift work.

Only interventions that have been tried and tested over many years are used, but only from treatment manuals for other disorders. For example: generalised anxiety [24], depression [25, 26], rumination [27], and positive psychology [28].

In addition, psychoeducational material was made available on healthy sleep, sleep disorders and their treatment [4–6, 29–31]. Information on shift work tolerance [32–34] and tips for dealing with shift work [35] were also included in the manual.

The use of these methods is not expected to cause harm to the participants. On the contrary, it is expected that these interventions will have greater positive effects on shift workers than classical CBT-I, because they can be better implemented for this target group.

The aim of the present study is to answer the following research question: Can the new insomnia therapy developed for shift workers (CBT-I-S) improve the sleep of shift workers?

### Explanation for choice of comparators {6b}

As already mentioned above, studies conducted to date using classic CBT-I methods have shown little effect on shift workers. A literature analysis and our own as yet unpublished study suggest that there are alternative factors maintaining sleep disorders in this group of people, which suggest that an alternative treatment option would be useful and more effective.

### Objectives {7}

To test these hypotheses, a newly developed insomnia treatment for shift workers (CBT-I-S) will be compared with the classical treatment of sleep disorders (CBT-I). The working hypothesis is that the experimental condition (CBT-I-S) will produce significant improvements in total sleep time, sleep onset latency, subjective sleep quality and daytime sleepiness in shift workers. These positive effects will be clearly superior to those of the standard condition (CBT-I) and will remain stable for 6 months after the end of treatment.

### Trial design {8}

The trial will be conducted in two parallel groups with controlled randomised allocation of the same number of subjects per group. The aim is to prove the superiority of the newly developed therapy (CBT-I-S) over standard therapy (CBT-I). Originally, an active control group was planned to keep a sleep diary for the same time period (8 weeks). Unfortunately, due to difficulties in recruiting subjects, this group had to be cancelled.

## Methods: participants, interventions, and outcomes

### Study setting {9}

Participants will be recruited in German-speaking countries, mainly Germany and Austria. Recruitment will cover both urban and rural areas. The study is not limited to specific occupations or sectors but includes all shift-working occupational groups. Due to the nationwide recruitment of subjects, treatments will be administered in an online setting.

### Eligibility criteria {10}

Included are people of all genders, aged 18 to 65, who work shifts for at least 30 h a week and suffer from primary insomnia. We excluded people with pre-existing physical conditions such as sleep apnoea, restless legs syndrome or chronic pain. We also excluded people with a history of mental disorders, namely severe affective or anxiety disorders, acute substance abuse disorders, and psychotic or schizo-affective disorders.

The assessment of the inclusion criteria by means of interviews and the implementation of the interventions will be carried out by psychology master students from the University of Salzburg, who already have clinical experience and have undergone specific training.

### Interventions {11a}

Treatments in both conditions are delivered online by MS Teams in groups of 12 people with one trainer per group. Both conditions receive seven sessions over 7 weeks of 90 min each. As a starting point, the CBT-I-S group receives reading information material 1 week before the first session. The standard CBT-I condition starts keeping a sleep diary 1 week before the first session.

The implementation will start when enough participants have been found for two parallel groups per condition, to be able to take an alternative appointment in the parallel group in case of scheduling problems.

#### CBT-I-S (experimental condition)

This group will receive psychoeducation 1 week in advance as self-study reading material. The sessions will have the following contents:

1. Introduction, discussion of reading material, derivation of the therapeutic rationale, attitude to shift work
2. Tips for shift work, positive activities, relaxation, daily structure
3. Problem solving, acceptance, resource orientation
4. (Depressive) rumination
5. Fear/anxiety/worry
6. Mood
7. Conclusion: exchange of experiences, emergency kit, farewell
  [4–6, 17, 25–35]

#### CBT-I (standard condition)

From 1 week before the first session until the last session, this group keeps a sleep diary. Session 1 to 7 will have the following contents:

1. Introduction, psychoeducation, relaxation
2. Introduction of sleep restriction, calculation of sleep window
3. Deepen sleep restriction, repeat relaxation
4. Stimulus control
5. Sleep hygiene
6. cognitive restructuring
7. Exchange of experiences, sleep protocols, relapse prevention, farewell
  [14, 29–31, 36, 37]

Sleep restriction is not easy to implement for shift workers, but should nevertheless be applied in the standard group, in a slightly adapted form: The reduction of total time in bed to total sleep time is emphasised as the main effective factor. In order not to jeopardise the compliance of shift workers, less emphasis is placed on the regularity of the sleep window.

### {11b}

The interventions will be stopped or modified if, contrary to expectations, the trainers notice a deterioration in individual participants during the sessions, or if participants contact the study management with complaints.

### {11c}

Participants are motivated to take an active part in the sessions. Attendance is monitored through homework, which is discussed during the sessions, and through the trainers’ assessment of participation in the group.

### {11d}

During the course of therapy, subjects should not receive any other treatment for sleep disorders. If someone is in therapy for other reasons, they may not need to be excluded. This must be assessed on an individual basis. Occasional use of sleeping pills is allowed.

### Outcomes {12}

The primary outcome is improvement in the following key sleep variables: total sleep time, sleep onset latency, subjective sleep quality and daytime sleepiness. We also want to measure the severity of the sleep disorder.

Secondary outcomes are improvements in depressive mood and anxiety, dysfunctional sleep attitudes, and cognitive and physiological arousal.

Data will be collected at three time points: before starting treatment, at its end and 6 months later as a follow-up. We will compare group means between groups and within groups at each time point. Since we will have multiple primary outcomes over multiple time points, we will adjust the α-level using a Bonferroni correction.

The magnitude of the differences, especially in the pre-post comparisons, should not only be statistically, but also clinically significant. This means, for example, that subjects no longer meet the criteria for a sleep disorder (DSM-5, [11]) after treatment.

### Participant timeline {13}

**Fig. 1:**
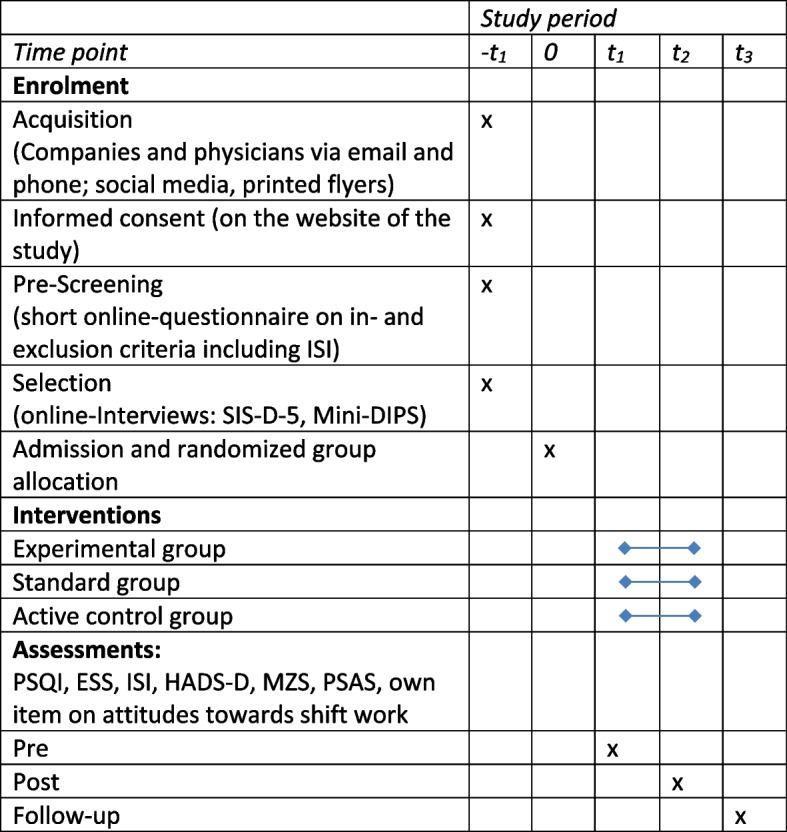
Participant timeline

See the participant timeline in Fig. 1. The period between *t*_1_ and *t*_2_ is 8 weeks, and the period between *t*_2_ and *t*_3_ is 6 months.

### Sample size {14}

To determine the required sample size, a power estimation was carried out with G*Power [38]: ANOVA with repeated measures and within-between interactions, 2 groups and 3 measurement times, small expected effect 0.1, desired power of 0.80 (1-β), *p* = 0.01 (4 outcome variables), correlation among repeated measures 0.7 results in a total sample size of *N* = 142.

### Recuitment {15}

We use a number of strategies to achieve the target sample size: We contact companies with shift work by email and ask them to forward our call for participation to their employees. As there has been little response so far to this strategy, we are now increasingly using telephone contact. We are also mailing to several thousand doctors in Germany and Austria: General practitioners, psychiatrists, and occupational physicians. Some of them have asked for printed flyers.

The study will also be promoted through social media (Facebook, Instagram). Printed flyers will also be distributed (pharmacies, personal contacts, etc.). The fact that participants will receive free treatment for their sleep problems does not seem to be enough of an incentive. That is why we are now offering a financial compensation of 50 euros per participant. We hope to get more feedback on this.

## Methods: assignment of interventions (for controlled trials)

### Allocation {16a-c}

To randomly assign subjects to groups, participants will be numbered consecutively by the study management in the order of their enrolment. The trainers use a computer-generated random number sequence and match these numbers to the participants’ sequential number sequence. The final allocation of participants to conditions is made by the study management.

There is allocation concealment, as only number sequences are used in the allocation process, and no one is able to assign a name to a number.

### Blinding {17a-b}

There is no blinding. Both trainers and participants know which group they are in.

## Methods: data collection, management, and analysis

### Data collection methods {18a}

The data collection is done online to avoid the influence of the evaluators. As we primarily use self-report questionnaires, socially desirable responses cannot be excluded. An attempt is made to minimise this bias by providing appropriate information in the questionnaire instructions. Missing data are avoided by making all questions mandatory.

Below is a description of the integrated questionnaires according to primary and secondary outcomes. The interpretation of Cronbach’s alpha values follows George and Mallery [39].

#### Primary outcomes

- PSQI: Pittsburgh Sleep Quality Index [40, 41]. This questionnaire measures sleep onset latency, total sleep time and subjective sleep quality. The re-test reliability is good, *r* = 0.82–0.89 [40], Cronbach’s *α* is also good with a value of 0.80 [41], the diagnostic validity is over 80% (sensitivity) or between 83 and 87% (specificity; quoted from [40]). So it can also be considered good.
- ESS: Epworth Sleepiness Scale [42], to measure daytime sleepiness. Cronbach’s α is acceptable to good with values between 0.73 and 0.88. According to Kendzerska et al. [43], re-test reliability is only described as good by one study with a value of *r* = 0.82, other studies found lower values. In their meta-analysis, Kendzerska et al. [43] found medium validity.
- ISI: Insomnia Severity Index [44], to assess severity of sleep disorder. According to [45], quality criteria are overall acceptable to good: Cronbach’s *α* = 0.74–0.78. In the opinion of these authors, further findings on concurrent validity and reliability are evidence that it is a valid and sensitive instrument.

#### Secondary outcomes

- HADS-D: Hospital Anxiety and Depression Scale [46] to measure anxiety and depressive mood. Test quality criteria are predominantly good: Internal consistency: Cronbach’s alpha, patient sample *α* = 0.80 (anxiety), *α* = 0.81 (depression). Re-test reliability: *r* = 0.69–0.89 (anxiety) or *r* = 0.85 (depression). Convergent validity: *r* = 0.54–0.77 [46, 47].
- MZS: Dysfunctional sleep attitudes (Opinions about sleep questionnaire, based on the Dysfunctional Beliefs and Attitudes about Sleep, DBAS-16; [48]), measures dysfunctional and irrational beliefs about sleep. Weingartz and Pillmann [48] state that the internal consistency is good, with a Cronbach’s alpha = 0.87, and they describe the validity and reliability as sufficient to good.
- PSAS: Pre-Sleep Arousal Scale [49]. Measures cognitive and physiological arousal in the 2 h before falling asleep. In a doctoral thesis by Vochem [50], the internal consistency is described with reference to the original [51] as follows “…for healthy sleepers on the physical arousal subscale as good (0.84) and on the cognitive arousal subscale as questionable (0.67),” “for insomnia patients as acceptable (0.76) and good (0.81).” ([50], p. 47). Retest reliability (3 weeks) is reported by the original authors [51] as *r* = 0.72 for the cognitive subscale and *r* = 0.76 for the somatic subscale.
- Own item: “Do you like working shifts?” to measure attitudes towards shift work.

### {18b}

In order to keep the dropout rate low, the trainers have to emphasise the importance of data collection in the group sessions. The subjects are first reminded of the measurement times by e-mail. If this is not sufficient, a phone call follows. The offer to take part in the most effective therapy after data collection is intended to provide an incentive to take part in the follow-up measurement. A further incentive for participants not to drop out of the study or the data collection prematurely is the 50 Euro compensation for effort, which will only be paid out at the end of the study.

### Data management {19}

As the data is collected online, no data entry is required. The subjects have already defined a subject code for themselves at the pre-screening stage, following precise instructions. This consists of the first letter of the mother’s first name, the first letter of the mother’s maiden name and the day of the mother’s birthday. This code is used to store and process the data. After pre-screening, no further names are stored with the data. The data will be collected using LimeSurvey and only exported to SPSS in pseudonymised form for processing.

Data quality is promoted through plausibility checks: For each variable, it is checked whether the participants’ answers are within a plausible range.

The raw data is stored both in LimeSurvey and in an exported SPSS file. In addition to a local PC, the processed data is stored on an external hard drive as a backup. The required retention period is 7 years.

### Statistical methods {20 a-c}

For analysing primary and secondary outcomes, we will use linear mixed-models:

We will make within-subject comparisons of the primary and secondary outcomes at the three time points before and after treatment and at follow-up. We will also make between-subject comparisons, i.e., a comparison of the three conditions at the three time points.

Mediator and moderator analyses of the moderation and mediation of the outcomes: No hypotheses about mediators and moderators will be formulated. These analyses are purely exploratory.

We expect low attrition at the post-measurement, but somewhat higher at the follow-up measurement. Individuals who did not complete the post-measurement will be excluded. Those who did not complete only the follow-up may be included in the pre-post comparison but must be excluded from the calculations of the stability of the effects. Depending on their number, it may be necessary to analyse whether they represent a group with specific characteristics. As no post- or follow-up data is expected to be available from the dropouts, an intention-to-treat analysis is not possible. Instead, a per-protocol analysis will be conducted with all participants who were present in at least 4 sessions.

## Methods: monitoring

### Data monitoring {21a-b}

We will not set up a Data Monitoring Committee (DMC) as we consider the risks to participants in this study to be minimal. Should any problems arise during the study, participants should contact their trainer or the study management. Based on these reports, the study management will decide whether the study should be modified or cancelled. The final decision will be made by the project supervisor. A formal interim analysis is not planned.

### Harms {22, 30}

Participants are asked to report any problems or impairments they experience as a result of the study to the trainer in their group or directly to the study management. They will be collected and, if necessary, discussed with the project supervisor.

We do not expect any harm to the participants. Should a negative development nevertheless occur in the course of the treatment, we will provide ancillary as well as post-trial care: We will work out possible solutions with the participants and, if necessary, offer alternative treatment options and appropriate contacts.

### Auditing {23}

During the delivery of treatment, the trainers are supervised separately according to the conditions. Supervision is provided by two experienced therapists who are independent of the trial management and sponsor.

### Protocol amendments {25}

An amendment to the protocol was submitted on 21 October 2023 to the Ethics Committee of the Paris-Lodron University of Salzburg to approve the cancellation of the control group and the payment of 50 Euro per participant. No further significant changes to the study protocol are expected. Therefore, there are no plans to communicate such changes to relevant parties.

### Confidentiality {27}

Personal information is collected, stored, and processed in a pseudonymous manner. Participants are helped to create their MS Teams account so that only their first name, not their last name, is visible to other participants. At the treatment’s beginning the trainers emphasise the need for confidentiality within the groups. Everyone involved in the project has signed a declaration of confidentiality regarding any personal information they become aware of.

### Dissemination policy {31 a-c, 33}

This study is part of a PhD project. It is therefore expected that the results of the study will be published in 2–3 papers in scientific journals. Participants can receive the results by e-mail on request.

The main authors of the study results are Tanja Grünberger (study management), Anton-Rupert-Laireiter (main supervisor), Manuel Schabus (co-supervisor), and Christopher Höhn.

It is not planned to use professional authors.

There are no plans for granting public access to the full protocol, participant-level data or statistical code. However, these data can be provided by the corresponding author upon request.

As this is a psychological therapy study, no biological samples will be collected. Accordingly, there are no plans to collect or store them.

## Discussion

### Strengths

The study design differs in many ways from other studies on shift work and sleep. First and foremost, the planned sample size of *N* = 142 will give the results more statistical power and thus significance. Due to the online implementation, the acquisition of participants does not have to be limited to the geographical area around the University of Salzburg but can be extended to the entire German-speaking area. In addition, all occupational groups with shift work are included, not just one occupational group, as is the case in many studies. This means that the results can be generalised to all shift workers.

Another strength is the design with two parallel groups: experimental and standard. The effectiveness of the new protocol can be directly compared with standard treatment. The applicability for shift workers and their acceptance can also be determined with this experimental design (drop-outs). Due to the difficulty of recruiting participants, there are several weeks between enrolment and the start of therapy for some participants. Although we do not have an explicit control group, we can still use the ISI data (pre-screening vs. pre-measurement) to check whether there has been spontaneous improvement without intervention.

The development of a specific manual can also be seen as a strength of this trail. Based on literature and the results of a preliminary study, factors that have a significant impact on the sleep of shift workers have been identified. Appropriate interventions were then selected and adapted. All interventions that had to do with regular schedules were removed in order not to jeopardise participant compliance.

However, what is innovative about this treatment manual is the implicit rationale for improving sleep. It is well known that an excessive focus on sleep disturbance can perpetuate the sleep disorder. However, most insomnia therapies focus on the disturbed sleep, thereby increasing the focus on the problem. In our manual, sleep is improved by reducing worry, anxiety, depressive rumination, etc. From the start of therapy, participants understand the key to better sleep: do not think about it and do not try to influence sleep.

### Limitations

A limitation of this study is that it is an online-only study. This decision was made under the impression of the corona pandemic, but also for purely practical reasons: 142 shift workers with sleep disorders are probably difficult to find in the Salzburg area, and face-to-face appointments plus travel could become a problem for shift workers. By opting for online delivery, we can extend the recruitment process nationally and save participants travel time.

However, there is a risk that less group cohesion will develop in this setting, especially if people come from completely different regions (e.g., North Rhine-Westphalia/Germany vs. Vorarlberg/Austria). There could be mentality and language problems here. The trainers are prepared for this during their training.

The inclusion of different professional groups has not only advantages. It is possible that the severity of the sleep disorder or the exact, individual symptoms differ between professions. An example is the police and fire services (sometimes traumatising) compared to manufacturing jobs (monotonous). This could affect both group dynamics and the effectiveness of therapy. The data need to be used to check whether subgroups need to be formed here.

Another limitation is the fact that the therapies are carried out by trained students. It would of course be better to use experienced psychotherapists with additional, specific training in sleep disorders. Unfortunately, this is not possible for financial reasons.

### Trial status

Protocol version 2, October 21, 2023.

Recruitment of participants began on July 13, 2023, and is expected to be completed by the end of the year.

## Data Availability

The study management and the project management will have access to the final trial data set. Pseudonymized data can be made available to other researchers upon request.

## Appendix

Informed consent materials {32}, see also study website [52].

## Abbreviations

CBT-I: Cognitive behavioural therapy for insomnia
CBT-I-S: Cognitive behavioural therapy for insomnia in shift workers
REM: Rapid eye movement (sleep stage)
DSM-5: Diagnostic and Statistical Manual of Mental Disorders
PSQI: Pittsburgh Sleep Quality Index
ESS: Epworth Sleepiness Scale
ISI: Insomnia Severity Index
HADS-D: Hospital Anxiety and Depression Scale
MZS: “Meinungen-zum-Schlaf-Fragebogen”
DBAS-16: Dysfunctional Beliefs and Attitudes about Sleep
PSAS: Pre-Sleep Arousal Scale

## References

[1] Crönlein T, Galetke W, Young P. Schlafmedizin 1x1. Berlin Heidelberg: Springer; 2017.

[2] Hermann E, Gassmann D, Munsch S. Schlafstörungen. In: Margraf J, Schneider S, editors. Lehrbuch der Verhaltenstherapie, vol. 2. Heidelberg: Springer Medizin Verlag; 2009 p. 187–224.

[3] Holzinger B, Klösch G. Schlafcoaching: Wer wach sein will, muss schlafen. Wien: Goldegg Verlag; 2013.

[4] Pollmächer T, Wetter TC, Bassetti CLA, Högl B, Randerath W, Wiater A, editors. Handbuch Schlafmedizin. München: Elsevier; 2020.

[5] Spiegelhalder K, Backhaus J, Riemann D. Schlafstörungen. 2nd ed. Göttingen: Hogrefe; 2011.

[6] Baglioni C, Espie CA, Riemann D, editors. Cognitive-behavioural therapy für insomnia (CBT-I) across the life span. Guidelines and clinical protocols for health professionals. Oxford: John Wiley & Sons Ltd; 2022.

[7] Reynolds AC, Sweetman A, Crowther ME, Paterson JL, Scott H, Lechat B, Wanstall SE, Brown BWJ, Lovato N, Adams RJ, Eastwood PR. Is cognitive behavioral therapy for insomnia (CBTi) efficacious for treating insomnia symptoms in shift workers? A systematic review and meta-analysis. Sleep Med Rev. 2023. 10.1016/j.smrv.2022.101716. 10.1016/j.smrv.2022.101716 36459948

[8] Nachreiner F, Arlinghaus A, Horn D. Unterschiedliche psychosoziale Effekte unterschiedlicher Schichtsysteme. Z Arb Wiss. 2019. 10.1007/s41449-018-00139-6.

[9] Costa G. Shift work and health: current problems and preventive actions. SH@W 2010; 10.5491/SHAW.2010.1.2.112. 10.5491/SHAW.2010.1.2.112 PMC3430894 22953171

[10] Seibt A, Knauth P, Griefahn B. Arbeitsmedizinische Leitlinie der Deutschen Gesellschaft für Arbeitsmedizin und Umweltmedizin e. V. Nacht- und Schichtarbeit. Arbeitsmedizin | Sozialmedizin | Umweltmedizin. 2006;41(8):390–7.

[11] Falkai P, Wittchen HU, editors. American Psychiatric Association: Diagnostisches und Statistisches Manual Psychischer Störungen: DSM-5 (2., korrigierte Auflage). Göttingen: Hogrefe; 2018.

[12] Riemann D, Morin CM, Reynolds CF. Das Kapitel Schlafstörungen im DSM-V – ein Zwischenbericht. Z Psychiatr Psychol Psychother. 2011;59(4):275–80.

[13] Drake CL, Roehrs T, Richardson G, Walsh JK, Roth T. Shift work sleep disorder: prevalence and consequences beyond that of symptomatic day workers. Sleep. 2004. 10.1093/sleep/27.8.1453. 10.1093/sleep/27.8.1453 15683134

[14] Espie CA. Standard CBT-I protocol for the treatment of insomnia disorder. In: Baglioni C, Espie CA, Riemann D, editors. Cognitive-behavioural therapy für insomnia (CBT-I) across the life span. Guidelines and clinical protocols for health professionals. Oxford: John Wiley & Sons Ltd; 2022. p. 19–41.

[15] Jang EH, Hong Y, Kim Y, Lee S, Ahn Y, Jeong KS, Jang T-W, Lim H, Jung E, Shift Work Disorder Study Group, Chung S, Suh S. The development of a sleep intervention for firefighters: the FIT-IN (Firefighter’s Therapy for Insomnia and Nightmares) Study. Int J Environ Res Public Health. 2020; 10.3390/ijerph17238738. 10.3390/ijerph17238738 PMC7727785 33255478

[16] Järnefelt H, Lagerstedt R, Kajaste S, Sallinen M, Savolainen A, Hublin C. Cognitive behavioral therapy for shift workers with chronic insomnia. Sleep Med. 2012(13); 10.1016/j.sleep.2012.10.003. 10.1016/j.sleep.2012.10.003 23168269

[17] Booker LA, Sletten TL, Barnes M, Alvaro P, Collins A, Chai-Coetzer CL, et al. The effectiveness of an individualized sleep and shift work education and coaching program to manage shift work disorder in nurses: a randomized controlled trial. J Clin Sleep Med. 2022. 10.5664/jcsm.9782. 10.5664/jcsm.9782 PMC8974377 34870586

[18] Atlantis E, Chow C-M, Kirby A, Singh MF. An effective exercise-based intervention for improving mental health and quality of life measures: a randomized controlled trial. Prev Med. 2004. 10.1016/j.ypmed.2004.02.007. 10.1016/j.ypmed.2004.02.007 15226056

[19] Lee KA, Gay CL, Alsten CR. Home-based behavioral sleep training for shift workers: a pilot study. Behav Sleep Med. 2014;12(6):455–68. 24229383 10.1080/15402002.2013.825840

[20] Ito-Masui A, Sakamoto R, Matsuo E, Kawamoto E, Motomura E, Tanii H, Yu H, Sano A, Imai H, Shimaoka M. Effect of an internet-delivered cognitive behavioral therapy-based sleep improvement app for shift workers at high risk of sleep disorder: single-arm, nonrandomized trial. JMIR. 2023. 10.2196/45834. 10.2196/45834 PMC10481224 37606971

[21] Ell J, Brückner HA, Johann AF, Steinmetz L, Güth LJ, Feige B, Järnefelt H, Vallières A, Frase L, Domschke K, Riemann D, Lehr D, Spiegelhalder K. Digital cognitive behavioural therapy for insomnia reduces insomnia in nurses suffering from shift work disorder: a randomized-controlled pilot trial. J Sleep Res. 2024. 10.1111/jsr.14193. 10.1111/jsr.14193 PMC11596998 38485134

[22] Vallières A, Pappathomas A, de Billy GS, Mérette C, Carrier J, Paquette T, Bastien CH. Behavioural therapy for shift work disorder improves shift workers’ sleep, sleepiness and mental health: a pilot randomized control trial. J Sleep Res. 2024. 10.1111/jsr.14162. 10.1111/jsr.14162 PMC11596990 38443322

[23] Espie CA, Broomfield NM, MacMahon KMA, Macphee LM, Taylor LM (2006). The attention-intention-effort pathway in the development of psychophysiologic insomnia: a theoretical review. Sleep Med Rev. 2006; 10.1016/j.smrv.2006.03.002. 10.1016/j.smrv.2006.03.002 16809056

[24] Becker E, Margraf J. Generalisierte Angststörung. Ein Therapieprogramm. Weinheim: Beltz-Verlag; 2002.

[25] Schaub A, Roth E, Goldmann U. Kognitiv-psychoedukative Therapie zur Bewältigung von Depression. Ein Therapiemanual (2. Aufl.). Göttingen: Hogrefe; 2013.

[26] Pitschel-Walz G, Bäuml J, Kissling W. Psychoedukation bei Depressionen. Manual zur Leitung von Patienten- und Angehörigengruppen. München: Urban & Fischer Verlag; 2003.

[27] Teismann T, Hanning S, Brachel R, Willutzki U. Kognitive Verhaltenstherapie depressiven Grübelns. Berlin: Springer; 2012.

[28] Feld A, Rudy JM. Coaching der Positiven Psychologie. Manual für Coaches. Salzburg: Paris-Lodron-Universität; 2017.

[29] Scharfenstein A, Basler H-D. Schlafstörungen. Auf dem Weg zu einem besseren Schlaf. Trainerhandbuch. Göttingen: Vandenhoeck & Ruprecht; 2004.

[30] Müller T, Paterok B. Schlaftraining. Ein Therapiemanual zur Behandlung von Schlafstörungen (2., überarbeitete Auflage). Göttingen: Hogrefe; 2010.

[31] Crönlein T. Primäre Insomnie. Ein Gruppentherapieprogramm für den stationären Bereich. Göttingen: Hogrefe; 2013.

[32] Reinberg A, Ashkenazi. Internal desynchronization of circadian rhythms and tolerance to shift work. Chronobiol Int. 2008; 10.1080/07420520802256101. 10.1080/07420520802256101 18622820

[33] Saksvik IB, Bjorvatn B, Hetland H, Sandal GM, Pallesen S. Individual differences in tolerance to shift work – a systematic review. Sleep Med Rev. 2011;15:221–35. 20851006 10.1016/j.smrv.2010.07.002

[34] Kerkhof GA. Shift work and sleep disorder comorbidity tend to go hand in hand. Chronobiol Int. 2018. 10.1080/07420528.2017.1392552. 10.1080/07420528.2017.1392552 29157012

[35] Järnefelt H, Spiegelhalder K. CBT-I Protocols for Shift Workers and Health Operators. In: Baglioni C, Espie C, Riemann D, editors. Cognitive-behavioural therapy for insomnia (CBT-I) across the life span. Guidelines and clinical protocols for health professionals. Chichester: John Wiley & Sons Ltd; 2022.

[36] Binder R, Schöller F, Weeß H-G. Therapie-Tools Schlafstörungen. Weinheim: Beltz; 2020.

[37] Scharfenstein A, Basler H-D. Schlafstörungen. Auf dem Weg zu einem besseren Schlaf. Schlaftagebuch. Göttingen: Vandenhoeck & Ruprecht; 2004.

[38] Faul F, Buchner A, Erdfelder E, Lang A-G, Buchner A. G*Power3: a flexible statistical power analysis program for the social, behavioral, and biomedical sciences. Behav Res Methods. 2007;39(2):175–91. 17695343 10.3758/BF03193146

[39] George D, Mallery P. SPSS for Windows step by step: a simple guide and reference (11.0 update, 4th ed.). Boston: Allyn & Baco; 2003.

[40] Buysse DJ, Reynolds CF, Monk TH, Berman SR, Kupfer DJ. The Pittsburgh Sleep Quality Index: a new instrument for psychiatric practice and research. Psychiatry Res. 1989. 10.1016/0165-1781(89)90047-4. 10.1016/0165-1781(89)90047-4 2748771

[41] Carpenter JS, Andrykowski MA. Psychometric evaluation of the Pittsburgh Sleep Quality Index. J Psychosom Res. 1998;45(1):5–13. 9720850 10.1016/S0022-3999(97)00298-5

[42] Johns MW. A new method for measuring daytime sleepiness: the Epworth sleepiness scale. Sleep. 1991. 10.1093/sleep/14.6.540. 10.1093/sleep/14.6.540 1798888

[43] Kendzerska TB, Smith PM, Brignardell-Petersen R, Leung RS, Tomlinson GA. Evaluation of the measurement properties of the Epworth sleepiness scale: a systematic review. Sleep Med Rev. 2014. 10.1016/j.smrv.2013.08.002. 24135493 10.1016/j.smrv.2013.08.002

[44] Morin CM. Insomnia: Psychological assessment and management. New York: Guilford Press; 1993.

[45] Bastien CH, Vallières A, Morin CM. Validation of the Insomnia Severity Index as an outcome measure for insomnia research. Sleep Med. 2001. 10.1016/s1389-9457(00)00065-4. 10.1016/s1389-9457(00)00065-4 11438246

[46] Herrmann-Lingen C, Buss U, Snaith RP. Hospital Anxiety and Depression Scale, Deutsche Version (HADS-D) (Vol. 3). Huber: Bern; 2011.

[47] Geue K, Strauß B, Brähler E, editors. Diagnostische Verfahren in der Psychotherapie. Göttingen: Hogrefe; 2016.

[48] Weingartz S, Pillmann F. Meinungen-zum-Schlaf-Fragebogen. Deutsche Version der DBAS-16 zur Erfassung dysfunktionaler Überzeugungen und Einstellungen zum Schlaf. Somnologie. 2009; 10.1007/s11818-008-0356-6.

[49] Gieselmann A, de Jong-Mayer R, Pietrowsky R. Kognitive und körperliche Erregung in der Phase vor dem Einschlafen. Die deutsche Version der Pre-Sleep Arousal Scale (PSAS). Z Klin Psychol Psychother. 2012;41(2):73–80. 10.1026/1616-3443/a000134

[50] Vochem JK. Wie beeinflussen körperliche und kognitive Erregung sowie nächtliches Auf-die-Uhr-Schauen den Schlaf? Normierung der Fragebögen Pre Sleep Arousal Scale (PSAS) und Time Monitoring Behaviour-10 (TMB-10) anhand von gesunden Schläfern und Anwendung bei Patienten mit primärer Insomnie. Inaugural-Dissertation zur Erlangung des Medizinischen Doktorgrades der Medizinischen Fakultät der Albert- Ludwigs- Universität Freiburg i.Br.; 2017.

[51] Nicassio PM, Mendlowitz DR, Fussell JJ, Petras L. The phenomenology of the pre-sleep state: the development of the pre-sleep arousal scale. Behav Res Ther. 1985; 10.1016/0005-7967(85)90004-X. 10.1016/0005-7967(85)90004-x 4004706

[52] Grünberger T. Study website. http://www.schlaf-und-schicht.com. Accessed 15 Nov 2023.

